# APATHY AND ANXIETY ARE RELATED TO POOR FUNCTION IN PERSONS WITH EARLY-ONSET ALZHEIMER'S DISEASE

**DOI:** 10.1093/geroni/igac059.1007

**Published:** 2022-12-20

**Authors:** Adele Crouch, Lauren Massimo

**Affiliations:** University of Pennsylvania, Philadelphia, Pennsylvania, United States; University of Pennsylvania, Philadelphia, Pennsylvania, United States

## Abstract

Neuropsychiatric symptoms are prevalent in persons with early-onset Alzheimer’s disease (EOAD) and may contribute to the inability to perform instrumental activities of daily living. We examined associations between frequently observed symptoms in persons with EOAD: apathy, anxiety, depression, and patient function. Caregivers of 94 persons with EOAD completed questionnaires including the Neuropsychiatric Inventory and the Functional Activities Questionnaire. Regression analyses were performed for each neuropsychiatric symptom as a predictor with covariates (age, sex disease duration) and our outcome was patient function. We then performed multivariate analysis with the significant predictors. We observed that apathy explained 20.51% [F(4,68)=5.65, adjusted R2=0.2051; p<0.001], anxiety explained 6.63% [F(4,70)=2.31, adjusted R2=0.0663 p<0.05], and depression was not a significant predictor of patient function. In a multivariate model, apathy and anxiety explained 21.03% [F(5,67)=4.83, adjusted R2=0.2103; p<0.001] of the variance in patient function. These results suggest apathy and anxiety contribute to diminished ability to complete functional activities.

